# Prevalence and associated factors of self-medication among pregnant women in N’Djamena North District, Chad

**DOI:** 10.11604/pamj.2026.53.39.48580

**Published:** 2026-01-28

**Authors:** Judicael Adadja, Bow Gamaou Allafi, Gregoire Falq, Carine Munezero, Fabrice Kadjogbe Odjarado, William Atade, Nafissatou Traore, Simon Berthelot

**Affiliations:** 1 Axe Santé des Populations et Pratiques Optimales en Santé, Centre de Recherche du Centre Hospitalier Universitaire de Québec, Université Laval, Québec, Canada,; 2 Institut de Formation et de Recherche Interdisciplinaire en Sciences de la Santé et de l'Éducation, Ouagadougou, Burkina Faso,; 3 Manson Unit, *Médecins Sans Frontières*, London, United Kingdom,; 4 École Internationale Mohamed VI de Santé Publique, Université Mohamed VI des Sciences et de la Santé, Casablanca, Maroc,; 5 Institut Régional de Santé Publique, Université d'Abomey-Calavi, Abomey-Calavi, Bénin

**Keywords:** Self-medication, pregnancy, pregnant women, prevalence, associated factors, determinants, N’Djamena Nord, Chad

## Abstract

**Introduction:**

self-medication during pregnancy is a common practice in many low- and middle-income countries, despite the associated risks for both mother and fetus. This study aimed to estimate the prevalence of self-medication and identify its associated factors among pregnant women in the N'Djamena North District of Chad.

**Methods:**

this was a multicenter cross-sectional study conducted among 348 pregnant women conveniently recruited from 15 public health centers in the N'Djamena North District. Data were collected through individual interviews using a structured questionnaire administered via KoboCollect. Univariate and then multivariable Generalized Estimating Equations (GEE) models were applied using a Poisson distribution and an exchangeable correlation structure to identify factors associated with self-medication. Statistical significance was set at 5%.

**Results:**

the prevalence of self-medication during pregnancy was 37.9% (95% CI: 32.8-43.0). Factors significantly associated with this practice included living in an urban area (Adjusted Prevalence Ratio [APR] = 1.54; 95% CI: 1.05 - 2.28), living in a polygamous household (APR = 1.21; 95% CI: 1.04-1.39), a history of self-medication (APR = 1.76; 95% CI: 1.36-2.28), and lack of knowledge about the associated risks (APR = 1.72; 95% CI: 1.39-2.12).

**Conclusion:**

self-medication is a common practice among pregnant women in the N'Djamena North District, driven by both structural and behavioural factors. These findings highlight the need to strengthen prenatal education and develop targeted prevention strategies to mitigate the risks associated with this practice.

## Introduction

Pregnancy is a particularly vulnerable physiological period during which women often experience various discomforts such as nausea, pain, or digestive disorders [1,2]. To relieve these symptoms, many pregnant women resort to self-medication [3]. According to the World Health Organization (WHO), self-medication refers to the use of medications by individuals to treat self-recognised symptoms, as well as the reuse of previously prescribed treatments without medical consultation [4]. Self-medication during pregnancy represents a global public health concern. A meta-analysis including 65 studies conducted across five continents estimated the global prevalence of self-medication during pregnancy at 44.5%, highlighting the magnitude of the phenomenon, particularly in low- and middle-income countries [2,3].

In Chad, a 2020 survey in private pharmacies in N'Djamena found that 31% of medicines were dispensed with a medical prescription, 40% at the direct request of clients, and 29% following advice at the counter, provided by pharmacists or, more often, by auxiliary staff [5]. This dispensing model, largely disconnected from regulatory oversight, makes medicines easily accessible without medical supervision or pharmaceutical expertise, thereby fostering and normalizing the practice of self-medication [6]. Although it may provide temporary relief for minor ailments, self-medication poses significant risks: misdiagnosis, inappropriate treatments, drug interactions, delays in proper medical care, and exposure to substandard or counterfeit products [7-9]. During pregnancy, these risks are compounded. On the one hand, self-medication may cause severe adverse effects for the mother, potentially leading to maternal death; on the other hand, it may jeopardize fetal health [7,10-12]. Many substances can cross the placental barrier and expose the fetus to teratogenic or toxic effects [7]. Potential complications include miscarriage, congenital anomalies, preterm birth, low birth weight, or perinatal death [10-15]. This situation is even more concerning as data on drug safety during pregnancy remain scarce, due to the systematic exclusion of pregnant women from clinical trials, leaving a substantial gap in clinical evidence [9]. This lack of knowledge turns every instance of self-medication into an uncontrolled risk in a context of shared uncertainty [16-18].

Given the persistently poor maternal and neonatal health indicators in Chad [19-21], it is crucial to document self-medication practices among pregnant women to inform public policies and health education strategies. To date, to the best of our knowledge, no study has addressed this issue in the country. This study therefore aims to fill this gap by estimating the prevalence of self-medication among pregnant women and identifying its associated factors in the N'Djamena North Health District of Chad.

## Methods

**Study design and setting:** this was a quantitative, multicenter cross-sectional study conducted between October and November 2023. It was carried out in the 15 first-level public health centers of the N'Djamena North Health District in Chad. This district is one of the five that make up the health delegation of the city of N'Djamena. Located in the 1^st^ arrondissement, in the northern part of the capital, it had an estimated population of 185,513 in 2020, with approximately 10,218 expected pregnancies, according to demographic projections by the Chadian Directorate of Statistics and Health Information Services (DSSIS) [21].

**Study population:** the study targeted pregnant women residing in the N'Djamena North Health District who attended antenatal care consultations at one of the 15 first-level public health centers in the district during the study period. Eligible participants were pregnant women aged 18 years or older who had provided free and informed consent to participate in the study. Pregnant women who presented obstetric complications, such as preeclampsia, antepartum hemorrhage, or severe anemia, were excluded, as assessed by the attending midwife or physician. Women were also excluded if they had a diagnosed mental disorder or cognitive impairment affecting their ability to consent, had hearing and/or speech impairments that interfered with the interview process, or did not understand either French or the main local languages spoken in the region.

**Sampling:** participants were selected using convenience sampling within each of the 15 included public health centers. Thus, all pregnant women who attended antenatal consultations on data collection days and met the eligibility criteria were consecutively enrolled in the study. The sample size was calculated using Cochran's formula for categorical data [22]: n = (Z^2^ x p x q) / d^2^, where p represents the expected prevalence of self-medication among pregnant women (31.5%), based on a previous study conducted in Nigeria [23], a neighboring country with similar contextual characteristics; q = 1 - p; Z is the standard normal value for a 95% confidence level (i.e., 1.96); and d is the margin of error, set at 5%. A 10% oversampling was applied to account for potential non-responses, resulting in a required sample size of 365 pregnant women.

**Data collection:** data were collected through face-to-face individual interviews using a structured questionnaire administered in digital format via the KoboCollect application [24]. Before its implementation, the tool was pretested among 20 pregnant women at a health facility located outside the N'Djamena North District to assess its clarity and make any necessary adjustments. The data collection team consisted of six experienced female interviewers supervised by a field coordinator. All team members received two days of training covering the study objectives, methodology, questionnaire content, and best practices in data collection. On collection days, interviewers introduced themselves to the health center staff and the midwives in charge of antenatal consultations, who facilitated contact with eligible pregnant women. These women were then approached individually by the interviewers, who explained the study, assessed eligibility, and obtained written informed consent before administering the questionnaire. Interviews were conducted in a quiet and private setting to ensure confidentiality and foster trust. Each interview lasted approximately 10 to 15 minutes. No identifying personal data was collected to preserve participants' anonymity. Refusal to participate had no impact on the quality of care received. The study received approval from the *Institut de Formation et de Recherche Interdisciplinaire en Sciences de la Santé et de l'Éducation (IFRISSE)*. Administrative authorizations were also obtained from health authorities, including the head of the N'Djamena North District and the directors of the participating health centers. No financial or material compensation was offered. Participants were informed of their right to decline or withdraw from the study at any time without affecting their participation in the study or their medical care.

**Studied variables:** the dependent variable was the practice of self-medication during the current pregnancy. It was a binary variable coded as yes/no. A response was coded as “yes” if the participant reported having used medicinal products at least once during her pregnancy without prior consultation with a healthcare professional, or if she stated having added a medication to a medical prescription on her own initiative without systematically informing the prescriber [4]. Only conventional pharmaceutical drugs were considered; traditional remedies such as medicinal plants, herbal decoctions, or homemade remedies were excluded. Independent variables were selected based on the literature and categorized into three main groups:

***Sociodemographic variables:*** age, marital status, family type, level of education, place of residence (urban/rural, as defined by the Ministry of Health), presence of the husband in the household, distance between home and the health center, participant's occupation, household income, and partner's occupation.

***Gynecological and obstetric variables:*** gravidity, gestational age at the time of the survey, number of antenatal care visits, occurrence of pregnancy-related illnesses, and types of conditions reported.

***Variables related to self-medication:*** history of self-medication and level of knowledge about the risks of self-medication during pregnancy (based on the spontaneous citation of at least 3 risks).

**Data processing and analysis:** at the end of data collection, the data were exported from the KoboToolbox platform [24]. After verification and cleaning, a descriptive analysis was performed according to whether participants reported self-medicating during their current pregnancy. Continuous variables were summarized using means and standard deviations, while categorical variables were described using absolute and relative frequencies. The prevalence of self-medication was calculated by dividing the number of pregnant women who reported engaging in self-medication by the total number of participants surveyed. To identify factors associated with self-medication, Generalized Estimating Equations (GEE) models were used, specifying a Poisson distribution and an exchangeable correlation structure. Health centers were treated as clustering units, accounting for the correlation between observations within the same center [25,26]. This approach provided prevalence ratios, which are more appropriate than odds ratios in the context of a high event prevalence and often more useful for public health recommendations [27,28]. The identification process began with univariate models, testing the association between the dependent variable and each explanatory variable separately. Variables with a p-value ≤ 0.25 were included in a multivariable model. Additionally, variables considered relevant based on the literature were retained, even if they did not meet this threshold. Final variable selection was performed using a backward stepwise method, comparing models based on the Quasi-likelihood Information Criterion (QIC) [25]. Variables with a p-value below 0.05 were considered significantly associated with the practice of self-medication. All analyses were conducted using R software, version 4.5.0 [29].

## Results

**Socio-demographic characteristics of participants:** a total of 365 pregnant women were approached across the 15 public health centers in the N'Djamena North District. Of these, 348 agreed to participate, yielding a participation rate of 95.3%. The mean age of participants was 24.6 ± 5.3 years, with 46.0% aged 25 years or older. Nearly two-thirds (62.9%) had received no formal education. In terms of employment, 75.3% of the women were unemployed. The monthly income of the majority (88.2%) was less than or equal to 29,000 FCFA XAF (1 USD ≈ 620 FCFA). Regarding marital status, 97.7% of participants were in a relationship, and 66.4% lived in monogamous households. In 62.6% of cases, the husband lived in the same household as the participant at the time of the survey, regardless of marital or family arrangement. Additionally, 36.2% of women reported having a partner with salaried or commercial employment. Finally, 80.8% of participants resided in urban areas, and 91.4% lived within 5 km of a health center. These characteristics are summarized in Table 1.

**Table 1:** descriptive and univariate analysis of sociodemographic characteristics by self-medication status among pregnant women in N'Djamena North District, Chad (N = 348)

Variables	Self-Medication			Univariate analysis		
	Total n (col %)	No n (row %)	Yes n (row %)	CPR*	95% CI**	p-value
**Age (years)†**	24.6±5.3	24.7±5.3	24.5±5.3	1.00	1.00–1.01	0.56
**Age group**						
18–19 years	59 (17.0)	35 (59.3)	24 (40.7)	1.00	-	-
20–24 years	129 (37.1)	84 (65.1)	45 (34.9)	0.87	0.73–1.04	0.12
25–45 years	160 (46.0)	97 (60.6)	63 (39.4)	0.97	0.76–1.24	0.80
**Education level**						
No formal education	219 (62.9)	128 (58.4)	91 (41.6)	1.00	-	-
Primary	18 (5.2)	16 (88.9)	2 (11.1)	0.25	0.03–1.92	0.18
Secondary	94 (27.0)	59 (62.8)	35 (37.2)	0.86	0.59–1.26	0.45
Higher education	17 (4.9)	13 (76.5)	4 (23.5)	0.56	0.25–1.24	0.15
**Participant's occupation**						
Trader/Civil servant	61 (17.5)	37 (60.7)	24 (39.3)	1.00	-	-
Student/Pupil	25 (7.2)	20 (80.0)	5 (20.0)	0.51	0.26–1.00	0.04
Unemployed	262 (75.3)	159 (60.7)	103 (39.3)	1.00	0.72–1.38	0.98
**Marital status**						
In a relationship	340 (97.7)	212 (62.4)	128 (37.6)	1.00	-	-
Not in a relationship	8 (2.3)	4 (50.0)	4 (50.0)	1.33	0.78–2.28	0.29
**Family type**						
Monogamous	231 (66.4)	151 (65.4)	80 (34.6)	1.00	-	-
Single-parent	8 (2.3)	4 (50.0)	4 (50.0)	1.46	0.84–2.54	0.19
Polygamous	109 (31.3)	61 (56.0)	48 (44.0)	1.27	1.05–1.53	0.01
**Presence of husband in the household**						
Yes	218 (62.6)	143 (65.6)	75 (34.4)	1.00	-	-
No	130 (37.4)	73 (56.2)	57 (43.8)	1.29	1.00–1.67	0.05
**Husband's occupation**						
Salaried/Trader	126 (36.2)	81 (64.3)	45 (35.7)	1.00	-	-
Farmer/Breeder	43 (12.4)	25 (58.1)	18 (41.9)	1.18	0.84–1.66	0.34
Worker/Technician	95 (27.3)	55 (57.9)	40 (42.1)	1.17	0.95–1.44	0.14
Informal worker	84 (24.1)	55 (65.5)	29 (34.5)	0.97	0.79–1.18	0.74
**Participant's monthly income (FCFA XAF)‡**						
≤ 29,000	307 (88.2)	192 (62.5)	115 (37.5)	1.00	-	-
30,000–49,000	31 (8.9)	18 (58.1)	13 (41.9)	1.11	0.80–1.56	0.53
≥ 50,000	10 (2.9)	6 (60.0)	4 (40.0)	1.09	0.47–2.51	0.85
**Place of residence**						
Rural	67 (19.3)	43 (64.2)	24 (35.8)	1.00	-	-
Urban	281 (80.7)	173 (61.6)	108 (38.4)	1.08	0.67–1.73	0.76
**Distance to health center**						
≤ 5 km	318 (91.4)	200 (62.9)	118.0 (37.1)	1.00	-	-
> 5 km	30 (8.6)	16 (53.3)	14.0 (46.7)	1.25	1.02–1.53	0.02

* Crude Prevalence Ratio. **95% Confidence Interval. †Mean. ±Standard deviation. ‡CFA XAF (1 USD ≈ 620 FCFA)

**Obstetric characteristics and perceptions related to self-medication:** among the 348 participants included in this study, 77.3% were multigravida, and 54.3% were in their second trimester of pregnancy. The average number of antenatal care (ANC) visits was 2.6 ± 1.4, with 73.6% of women having completed no more than three ANC visits. In total, 72.4% of the women reported having experienced an illness during their current pregnancy, the most frequently reported conditions being malaria and typhoid fever (43.7%), followed by lower back and pelvic pain (21.8%). Additionally, 44.5% of participants reported having practiced self-medication in the past. About 66.1% stated they were aware of the potential consequences of this practice, while 26.2% expressed an intention to self-medicate in the future. The main reasons given for this intention included economic factors (44.0%, citing lack of financial means or the lower cost of medications), ease of access to drugs (17.6%), habit (11.0%), perceived effectiveness (11.0%), and distance from health facilities (8.8%). These findings are summarized in Table 2.

**Table 2:** descriptive and univariate analysis of obstetric characteristics and perceptions by self-medication status among pregnant women in N'Djamena North District, Chad (N = 348)

Variables	Self-medication			Univariate analysis		
	Total n (col %)	No n (row %)	Yes n (row %)	CPR*	95% CI**	p-value
**Gravidity**						
Multigravida	269 (77.3)	164 (61.0)	105 (39.0)	1.00		
Primigravida	79 (22.7)	52 (66.8)	27 (34.2)	0.86	0.57–1.29	0.46
**Gestational trimester**						
Third trimester	114 (32.8)	70 (61.4)	44 (38.6)	1.00		
Second trimester	189 (54.3)	112 (59.3)	77 (40.7)	1.05	0.87–1.28	0.61
First trimester	45 (12.9)	34 (75.6)	11 (24.4)	0.64	0.38–1.06	0.08
**Number of ANC**† **visits**						
≤ 3 visits	256 (73.6)	165 (64.5)	91 (35.5)	1.00		
> 3 visits	92 (26.4)	51 (55.4)	41 (44.6)	1.24	0.97–1.58	0.08
**Pregnancy-related illness**						
No	96 (27.6)	55 (57.3)	41 (42.7)	1.00		
Yes	252 (72.4)	161 (63.9)	91 (36.1)	0.84	0.66–1.08	0.18
**History of self-medication**						
No	193 (55.5)	138 (71.5)	55 (28.5)	1.00		
Yes	155 (44.5)	78 (50.3)	77 (49.7)	1.75	1.37–2.23	<0.001
**Knowledge of self-medication risks**						
Yes	230 (66.1)	160 (69.6)	70 (30.4)	1.00		
No	118 (33.9)	56 (47.5)	62 (52.5)	1.77	1.40–2.23	<0.001

* Crude Prevalence Ratio **95% Confidence Interval. † Antenatal Care

**Prevalence of self-medication among pregnant women:** out of the 348 participants surveyed, 132 reported having used self-medication during their current pregnancy, corresponding to a prevalence of 37.9% (95% CI: 32.8-43.0). Among these 132 women, 43.2% stated that they used self-medication in addition to a prescribed treatment, mainly due to perceived inefficacy of the prescribed drugs, high medication costs, or the occurrence of other concurrent illnesses. The remaining participants reported using self-medication exclusively. The medications were obtained from markets (55.5%), from friends or relatives (23.4%), or from pharmacies (22.1%).

**Identification of factors associated with self-medication among pregnant women:** following the univariate analyses (Table 1 and Table 2), 13 variables were retained for the development of the initial multivariable model. After variable selection, four factors were found to be significantly associated with the practice of self-medication among pregnant women: place of residence, family type, history of self-medication, and knowledge of the risks associated with this practice (Table 3). Pregnant women living in urban areas had a 1.54 times higher prevalence of self-medication than those in rural areas (APR = 1.54; 95% CI: 1.05-2.28; p = 0.03). Likewise, women from polygamous households showed a 1.21 times higher prevalence than those from monogamous households (APR = 1.21; 95% CI: 1.04-1.39; p = 0.01). Moreover, women who had practiced self-medication before pregnancy had a 1.76 times higher prevalence compared to those without such a history (APR = 1.76; 95% CI: 1.36-2.28; p < 0.001). Finally, participants who were unaware of the risks associated with self-medication had a 1.72 times higher prevalence than those who were aware of these risks (APR = 1.72; 95% CI: 1.39-2.12; p < 0.001).

**Table 3:** final multivariable model of factors associated with self-medication among pregnant women in N'Djamena North District, Chad (N = 348)

Variables	Self-Medication			Multivariable analysis		
	Total n (col %)	No n (row %)	Yes n (row %)	APR*	95% CI**	p-value
**Place of residence**						
Rural	67 (19.3)	43 (64.2)	24 (35.8)	1.00		
Urban	281 (80.7)	173 (61.6)	108 (38.4)	1.54	1.05−2.28	0.028
**Family type**						
Monogamous	231 (66.4)	151 (65.4)	80 (34.6)	1.00		
Single-parent	8 (2.3)	4 (50.0)	4 (50.0)	1.39	0.92−2.09	0.12
Polygamous	109 (31.3)	61 (56.0)	48 (44.0)	1.21	1.04−1.39	0.01
**History of self-medication**						
No	193 (55.5)	138 (71.5)	55 (28.5)	1.00		
Yes	155 (44.5)	78 (50.3)	77 (49.7)	1.76	1.36−2.28	<0.001
**Knowledge of self-medication consequences**						
Yes	230 (66.1)	160 (69.6)	70 (30.4)	1.00		
No	118 (33.9)	56 (47.5)	62 (52.5)	1.72	1.39−2.12	<0.001

* Adjusted Prevalence Ratio **95% Confidence Interval

## Discussion

This study aimed to estimate the prevalence of self-medication and identify its associated factors among pregnant women attending public health centers in the N'Djamena Nord Health District of Chad. It revealed that just over one-third of participants (37.9%) engaged in self-medication during pregnancy. Four factors were found to be significantly associated with this practice: place of residence, family type, history of self-medication, and knowledge of the risks associated with the practice.

The observed prevalence, relatively high, occurs within a regulatory context governed by Law No. 024/PR/2000 of November 24, 2000, which oversees pharmaceutical distribution in Chad. This suggests shortcomings in the effective enforcement of the legal framework. The results are comparable to those reported in other low- and middle-income countries, such as Ethiopia (33.9%), Nigeria (31.5%), Morocco (32.0%), and Brazil (36.0%) [23,30-32]. A meta-analysis [2] based on studies conducted in Iran, Tanzania, Nigeria, Ethiopia, and China reported an average prevalence of 32.0%. In contrast, lower prevalence rates have been reported in India (8.5%), Baghdad (11.0%), Indonesia (11.0%), France (22.0%), and Yemen (24.9%) [16,33-36], while higher rates were found in Ghana (65.5%) and Iran (49.1%) [37,38]. These variations may reflect structural and cultural differences between countries, as well as methodological differences between studies, particularly regarding the reference periods, study settings, or definitions of self-medication. In this study, only conventional pharmaceutical drugs were considered, excluding traditional remedies such as medicinal plants, herbal decoctions, or home remedies, unlike other studies that included them.

One of the most striking findings concerned place of residence. Contrary to several studies from Iran and Ethiopia that reported higher prevalence in rural settings [37-40], women living in urban areas of N'Djamena were more likely to self-medicate. This discrepancy may be explained by greater exposure to informal drug distribution channels in urban centers, where unauthorized vendors, locally known as “docteurs tchoukou”, are common, as well as easier access to unregulated medications, particularly through local markets [41,42]. Additionally, the circulation of unverified information, often shared by family or non-medical sources, may increase urban women's confidence in managing mild symptoms themselves, reducing their use of formal healthcare services.

Family structure also emerged as a key determinant. Women living in polygamous households practiced self-medication more frequently than those from monogamous families. This trend could reflect resource fragmentation and limited support within polygamous households, as well as a higher degree of autonomy in health-related decision-making. Furthermore, the cohabitation of multiple wives in the same household may facilitate the exchange of advice or medications, contributing to the normalization and trivialization of self-medication.

A prior history of self-medication appeared to be a strongly associated factor. Women who had previously engaged in self-medication were nearly twice as likely to do so again during pregnancy. This suggests that past experiences influence the development of self-care behaviors perceived as effective, accessible, or sufficient to manage minor symptoms. This observation is consistent with several studies conducted in Serbia, Iran, and Ethiopia [31,37,40,43-47].

Lastly, lack of awareness regarding the risks of self-medication also proved to be an important factor. Participants who were unaware of such risks were significantly more likely to self-medicate, a finding that aligns with results from Niazi *et al*. in Baghdad, Sarani *et al*. in Iran, and Ahmed *et al*. in Ethiopia [35,48,49]. This highlights the need to strengthen health education efforts during antenatal consultations, adapting messages to patients' levels of health literacy and explicitly addressing risks such as teratogenic effects, drug interactions, and symptom aggravation.

This study presents several strengths, including exhaustive coverage of all 15 health zones in the district, the use of a digital data collection tool that minimized data entry errors, and the involvement of experienced female interviewers who fostered a trusting environment in a culturally sensitive setting. The use of GEE models with a Poisson distribution allowed for the clustering structure of the data to be accounted for, producing robust estimates [25,26].

However, some limitations must be considered. The cross-sectional design does not allow for causal inference. The use of convenience sampling, a non-probability method, introduces a risk of selection bias. The exclusion of private healthcare facilities and women who do not attend antenatal care limits the generalizability of the findings to all pregnant women in the district. It is also possible that some women declined to participate due to their self-medication behaviors, potentially leading to either an underestimation or overestimation of the true prevalence. However, the observed refusal rate was relatively low (4.7%) and had been anticipated at 10% in the sample size calculation.

Finally, as data were collected via self-report, there is a risk of recall bias, particularly for questions requiring participants to remember past events. In addition, the negative perception often associated with self-medication may have led some women to conceal their practices, introducing social desirability bias. To minimize these information biases, interviews were conducted in settings that ensured confidentiality, and the interviewers were specifically trained to build rapport and foster a climate of trust. Questions were repeated if necessary, and participants were given adequate time to respond. Despite these limitations, the results of this study are consistent with existing literature and provide relevant insights to guide targeted interventions aimed at preventing the risks associated with self-medication during pregnancy in similar contexts.

## Conclusion

This study revealed a prevalence of 37.9%, indicating that more than one-third of pregnant women in the N'Djamena North District engaged in self-medication. Several factors were associated with this practice, including place of residence, family type, history of self-medication, and awareness of the associated risks. These findings provide a valuable basis for generating hypotheses regarding the determinants of self-medication and justify the need for further research to better understand this phenomenon. They also highlight the importance of implementing targeted prevention strategies and strengthening health policies to reduce self-medication among pregnant women.

### What is known about this topic

- Self-medication during pregnancy is a common practice worldwide, particularly in low- and middle-income countries, and is recognized as a public health concern;
- The consequences of self-medication in pregnant women can be severe, including fetal risks (malformations, miscarriages) and maternal risks (complications, delayed appropriate care);
- Easy access to medicines without prescription and weak regulatory control promote this practice in many African countries.

### What this study adds

- The prevalence of self-medication among pregnant women in N'Djamena Nord health district of Chad is 37.9%;
- More than one-third of the women surveyed were unaware of the risks of self-medication for their own health and that of the fetus;
- Four key factors were identified as being associated with this practice, including household type (monogamous, polygamous or single parent), a factor that has rarely been explored in this context.
